# Risk Factors for Postoperative Donor Site Complications in Radial Forearm Free Flaps

**DOI:** 10.3390/medicina60091487

**Published:** 2024-09-12

**Authors:** Seungeun Hong

**Affiliations:** Department of Plastic and Reconstructive Surgery, Ewha Womans University Seoul Hospital, College of Medicine, Ewha Womans University, 1071 Gonghangdae-ro 260, Gangseo-Gu, Seoul 07985, Republic of Korea; monkeyhong@ewha.ac.kr

**Keywords:** radial forearm flap, donor site complication, hypertension, acellular dermal matrix

## Abstract

*Background and Objectives:* The radial forearm free flap (RFFF) is the most commonly used flap for head and neck reconstruction. However, complications at the donor site are its major drawbacks. We aimed to identify the patient comorbidities and factors that predict donor site complications after RFFF. *Materials and Methods:* A retrospective chart review of consecutive patients who underwent RFFF reconstruction for head and neck cancer between 2015 and 2022 was performed. Demographic variables, clinical processes, and postoperative complications were assessed. All variables were analyzed using univariate and multivariate analyses. *Results:* Sixty-seven patients underwent RFFF reconstruction, and all received a split-thickness skin graft at the donor site. Twenty-five patients experienced delayed skin graft healing, whereas nine experienced sensory changes at the donor site. Hypertension and age had statistically significant negative effects on wound healing. The incidence of hand swelling was related to graft size, and the occurrence of paresthesia was significantly higher in diabetic patients and significantly lower in those with acellular dermal matrix (ADM). *Conclusions:* Patients with hypertension had a higher risk of prolonged wound healing after RFFF than their normotensive patients. Clinicians should pay particular attention to wound healing strategies in patients with hypertension. Additionally, better neuropathy care is recommended to achieve sensory recovery after RFFF in patients with diabetes. Using a skin graft with ADM could be a method to alleviate neurological symptoms.

## 1. Introduction

Choosing a flap is critical in the reconstruction of soft tissue defects after the excision of head and neck cancers. Since the head and neck are three-dimensional structures with various functional and cosmetic features, these factors should be considered when selecting a flap. Therefore, thin-textured and pliable flaps that facilitate folding are preferred in many cases, such as intraoral and extraoral soft tissue defects in the head and neck, and free flap reconstruction is the treatment of choice. The radial forearm free flap (RFFF) is a representative flap that satisfies these conditions. Since its introduction, it has become particularly popular and accepted worldwide for the reconstruction of soft tissue defects, such as those of the tongue or intraoral tissues. Despite the several advantages of the RFFF, it has lost ground in favor of the anterolateral tight flap (ALTF) and other free perforator flaps [1] because of the high rate of donor site morbidity when it is not possible to close the wounds primarily and skin grafts are needed [2]. The donor site on the forearm is a visible anatomical region with high mobility and functional importance.

Although the clinical indications for soft tissue head and neck reconstruction using ALTFs have increased, ALTFs are not always suitable for all patients. Because of the variable vascular anatomy and complications in patients with obesity, these flaps contain excess adipose tissue for reconstruction. Excessive fat tissue can lead to insufficient blood supply to the skin, and flap thinning can pose a threat [3,4]. Owing to these drawbacks, the use of the RFFF has been considered in some cases. Moreover, its advantages, such as thinness and good skin quality on a long vascular pedicle of large-diameter vessels, have led to RFFF survival rates exceeding 95%; hence, it can be useful even in revision surgery. In the case of head and neck cancer, which is particularly prevalent in older individuals, patients may have less resistance to RFFFs for reconstruction because of the characteristics of the elderly, who are less sensitive to scars. Therefore, in the assessment of scars, which are a representative complication of the RFFF, subjective indicators, such as how much discomfort the patient feels and whether he or she wants treatment, should be given more significance than simply evaluating the scar as an objective indicator. RFFFs must be used continuously, and postoperative donor site complications remain a major concern in patients undergoing RFFF. Therefore, several studies have attempted to address donor site morbidity [5,6]. Studies have focused on scarring and wound-healing problems. However, patients sometimes consider that newly proposed methods result in unsatisfactory outcomes. It remains unclear whether morbidity at the donor site is caused by raising the flap itself or by the closure of the donor site.

Ferrari et al. reported a correlation between the diminished functional capacity of vital organs in older patients and perioperative and postoperative morbidity and mortality [7]. In another study on microvascular-free tissue transfer in older patients, old age was a relative contraindication, and this increased risk was attributed to an increased likelihood of underlying medical problems in older patients, including hypertension, ischemic heart disease, pulmonary disease, and diabetes [8]. In other words, a careful selection of older patients based on comorbidities and general medical conditions is one of the most important ways to reduce postoperative complications, thereby improving surgical outcomes. Recognizing the risk factors for increased donor site complications after RFFF reconstruction may allow for better operative planning and more accurate anticipation of operative outcomes. As the RFFF is widely used in older patients, individual factors may affect the occurrence of donor site complications. Therefore, risk factors should be identified, and prevention of these factors is critical for management. To our knowledge, no studies have focused on the factors presenting as risk factors for radial forearm donor site complications in older patients. This study aimed to comprehensively review the risk factors that serve as independent predictors of postoperative donor-site complications after RFFF reconstruction for head and neck cancer. 

## 2. Materials and Methods

### 2.1. Study Design

**Figure 1:**
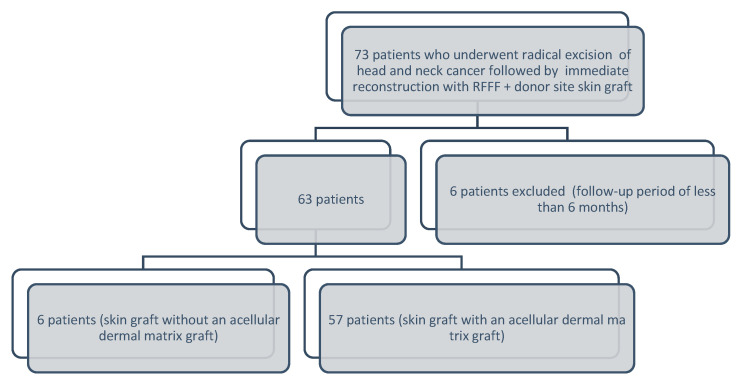
Flowchart depicting the patient selection and study design.

After receiving approval from our institutional review board (approval number 17-1347), we performed a retrospective analysis of patients who underwent radical excision of head and neck tumors (including ipsilateral or bilateral neck dissection), underwent immediate reconstruction with RFFF, and required a skin graft for the donor site with or without an acellular dermal matrix (ADM) graft (between January 2015 and December 2022). Patients with follow-up periods of less than 6 months were excluded (Figure 1). All patients provided written informed consent prior to surgery. Patient demographics, medical history, clinical variables, tumor factors, surgical history, skin graft site size (cm^2^), ADM use, arm direction used in the RFFF, and outcomes at each donor site were investigated by retrospectively reviewing electronic medical records and clinical photographs. These factors were analyzed independently to determine whether their presence was associated with postoperative donor site complications after RFFF reconstruction for head and neck cancers. Cosmetic results were evaluated using a questionnaire and careful clinical examination.

### 2.2. Preoperative Evaluation

In the sitting position, arterial blood flow to the hands was examined using the modified Allen test. Both hands were tested for comparison. Preoperatively, all patients were assessed for RFFF harvesting using handheld Doppler and computed tomography. If normal circulation was confirmed in both upper extremities, the non-dominant arm was chosen as the flap donor site for all patients.

### 2.3. Surgical Technique

Resections were performed by a head and neck surgeon, and defect reconstruction was performed by an experienced plastic surgeon to maintain consistency among the RFFF techniques, drain placement, and postoperative wound management. In all patients, flap dissection was performed using a tourniquet, and general anesthesia was used in all operations. All flaps were harvested as adipocutaneous flaps through suprafascial dissection, and the donor site was reconstructed with a split-thickness skin graft (STSG, 0.008–0.010-inch-thick) taken from the thigh on the same side as the flap, which was elevated using an electric dermatome (Zimmer Air Dermatome; Zimmer Inc., Warsaw, IN, USA). If the patient’s consent was obtained, three types of dermal matrixes (CGDerm^®^; CGBio, Inc., Seungnam, Republic of Korea/MegaDerm^®^; L&C Bio, Seoul, Republic of Korea/MatriDerm^®^; MedSkin Solutions Dr. Suwelack AG, Billerbeck, Germany) were randomly co-grafted at the donor site with the STSG. Several slit incisions were made on the harvested skin using a blade in the skin graft-only and skin grafted with ADM groups. The skin was fixed to the recipient bed using a stapler. All patients received postoperative perioperative antibiotic prophylaxis and wrist splinting for immobilization. 

### 2.4. Postoperative Wound Care

Negative pressure wound therapy (NPWT) (CuraVAC^®^; CGBio, Inc., Seungnam, Republic of Korea) was applied to all the skin graft sites in continuous mode at 125 mmHg. The dressing was changed 5 days postoperatively for the first time and then every other day, and the splint was applied for 7 days. The wound was examined for hematoma or seroma formation on the fifth postoperative day. Any fluid collected underneath the graft was aspirated using an 18-gauge needle. NPWT was maintained for up to 2 weeks, after which foam dressings were applied depending on the wound conditions until complete healing was achieved. 

### 2.5. Assessment of Outcomes

Early outcomes included whether hematoma or seroma occurred the first time the dressing was changed at the skin graft site, delayed wound healing (>3 weeks to complete healing, partial graft loss, tendon exposure, etc.), or complications (infections, wound dehiscence, and large skin loss that led to reoperation). Late outcomes were evaluated after a minimum of postoperative 6 months. Subjective assessments of persistent wrist stiffness, hand swelling, reduced hand strength, and changes in sensory function (paresthesia) were performed by the patient and physician and were compared with those of the contralateral wrist. The treatment history for scars, patient symptoms (itching and pain), and scar condition were also evaluated [9].

### 2.6. Statistical Analysis

The Wilcoxon rank-sum test was used to compare continuous variables according to the occurrence of complications. The association between categorical variables and the occurrence of complications was analyzed using Fisher’s exact test. Univariate analysis was performed to analyze the effect of each clinical variable on the occurrence of complications. Multivariable binary logistic regression was performed using all variables satisfying a value of *p* < 0.1 in the univariable analysis to analyze the independent effect of each clinical variable on the occurrence of complications. Data were expressed as median (interquartile range) for continuous variables. Categorical variables were expressed as numbers and percentages. All statistical analyses were performed using IBM SPSS for Windows software Version 25.0 (IBM Corp, Armonk, NY, USA). Statistical significance was defined as *p* < 0.05.

## 3. Results

### 3.1. Demographic and Clinical Variables

Sixty-seven consecutive patients (49 men and 18 women) with a median age of 66 (interquartile range, 52–79) years underwent radial forearm microvascular flap reconstruction after head and neck tumor ablation. Tongue cancer was the most common reason for surgery, followed by buccal cancer. The preoperative clinical history and physical examination revealed that 15 patients had hypertension, 22 had normotension, and 11 had diabetes mellitus. Patient demographics and clinical data are presented in Table 1. 

### 3.2. Surgical Variables

The microsurgical success rate was 100%. Re-exploration, classified as a major flap complication, was needed in one case, but no flap loss was observed. Minor flap complications were observed in three cases, including wound dehiscence and hematoma. The arm used as the donor site was the side other than the dominant arm. However, patient preference was also considered, and the left side was used in 52 patients (77.6%). Primary suturing at the donor site was not possible; therefore, a STSG was used in all cases. Three types of ADM were administered to 61 patients (91%) (Table 2). 

Total skin loss was not observed at the graft site. However, delayed skin graft healing, which was the most prevalent donor site complication, occurred in 25 patients. Among these, tendon exposure occurred in six patients (8.96%). Nevertheless, all patients were treated with conventional wound dressings without requiring further intervention. The most common late complication was paresthesia. Table 3 lists the donor site assessments. According to the Wilcoxon rank-sum and Fisher’s exact tests, age (*p* = 0.005) and hypertension (*p* = 0.005) were associated with delayed wound healing. Additionally, paresthesia was associated with both diabetes and the use of ADM (Table 4). 

### 3.3. Univariate Analysis for Risk Factors of Complications 

The results of the univariate analysis suggested that of the factors included in this study, preoperative accompanying diseases, including hypertension and diabetes, age, and ADM use were significantly associated with complications (*p* < 0.05). Among the demographic factors analyzed, body mass index, smoking history, and sex were not significant predictors of complications. Lymph node metastasis, chemotherapy, and length of hospitalization did not correlate with complications.

Hypertension (OR = 9.000, *p* = 0.005) and age (OR = 1.187, *p* = 0.009) were also significantly associated with delayed wound healing. In contrast, diabetes mellitus or ADM use and grafted skin size were not significantly associated with delayed wound healing. Additionally, no factors had a specific relationship with the occurrence of acute complications, such as seroma or hematoma. The occurrence of hand swelling was correlated with the skin graft area (OR = 1.047, *p* = 0.05). The incidence of paresthesia was high in patients with diabetes (OR = 13.714, *p* = 0.029). However, when ADM (OR = 0.071, *p* = 0.016) was used during skin grafting, the prevalence of paresthesia decreased (Table 5 and Table 6). No significant differences were observed according to the ADM type. In addition, none of the factors showed a specific relationship with other late complications.

Regarding scars, itching (score 3.7) rather than pain (score 2.3) was identified as a problem. Although the patients seemed to be more concerned about irregularity than the color, stiffness, and thickness of the scar, only three patients received scar-related treatment for this reason, and all of them were women (*p* = 0.007).

## 4. Discussion

Defects in the head and neck can be classified into six anatomical subareas (intraoral, mandibular, midfacial, cranial, cutaneous, and scalp) for reconstructive considerations [10]. Oral defects, such as those of the tongue and pharynx, can lead to functional deficits in speaking and swallowing depending on the site and extent of the lesion. Thin and pliable flaps are needed in this area because of the limited space and the need to maintain tongue mobility. As such, it is important to cover the soft tissue and to restore function through the reconstruction of the three-dimensional structure. Despite several local flap options, the microsurgical free flap is the accepted standard of care for head and neck reconstruction after tumor excision. This has enabled head and neck surgeons to perform more aggressive tumor resection. Currently, the workhorse soft tissue flap is the anterolateral thigh flap (ALTF). This perforator flap has the advantage of less donor site morbidity and allows for the versatility of harvesting variable amounts of soft tissue from various components. However, the ALTF is known for its anatomical variations. Although very rare, there may be cases in which the ALTF does not have a sizable skin vessel. Therefore, decisions regarding flap selection between ALTF and other flaps should consider the recipient and donor site characteristics, surgeon familiarity, and patient factors, such as body habitus and comorbidities.

Since its introduction by Yang et al. [10] in 1981, the RFFF has been used extensively for the reconstruction of head and neck defects owing to several distinct advantages. It is the best option when a thin, pliable flap with a long pedicle and sizable caliber is needed [11]. The pliability and thinness of the flap make it suitable for the reconstruction of complex three-dimensional defects. Furthermore, its attractiveness is enhanced because it is a reliable donor site, with flap failure rates reported to be less than 3%, and an anatomical location that permits a two-team harvest [12,13,14]. In our practice, the RFFF is the first option when reliability and safety are the primary considerations, such as in situations of previously failed free flaps, in older patients, or in those unable to endure long-term surgery. Nevertheless, there are potential morbidities associated with this flap harvest [15], which may lead surgeons to search for alternative free flaps with lower donor site morbidity. These may include wound healing disturbances with alterations in the range of movement, strength, and sensation in the donor’s forearm and cosmetically displeasing hypertrophic scars.

Several techniques have been developed for donor site closure after flap harvest. Skin grafts are required when it is not possible to close the primary wound, as in the case with other free flaps. The most common technique is the STSG, which is usually performed on the anterior thigh. However, disadvantages include decreased functional and aesthetic outcomes at the radial donor site, particularly in high-volume defects. Unfortunately, the STSG may be accompanied by morbidity due to impaired wound healing, such as partial graft loss, tendon exposure, or dehiscence. Tendon exposure can be caused by disruptive skin forces under the graft due to the continuous movement of the tendon [16]. Additionally, the use of full-thickness skin grafts did not improve the results. To avoid these wound healing problems, flexor tendons, particularly the flexor carpi radialis tendon, should be covered with bellies from the surrounding flexor muscles, particularly when a STSG is used [10]. The muscular cover creates a well-perfused wound bed for the ingrowth of the STSG, and the muscle directly protects the tendons and prevents their exposure. Nevertheless, despite applying the same surgical method to all patients, wound healing impairments occur; therefore, research on the factors affecting these impairments is necessary.

The treatment of wound healing impairments at the donor site is typically long and burdensome and often requires additional procedures, which may prolong hospital stays and reduce the patient’s quality of life. Delayed wound healing is a risk factor for various complications and must be avoided. Several studies have investigated methods to reduce these disturbances and have demonstrated improvements if the wound bed is optimized by enhancing granulation and capillary sprouting as preconditions for graft survival [17]. The presence of medical comorbidities as a predictor of complications in the free-flap reconstruction of the head and neck has been described in several studies. Nevertheless, few studies have investigated the predictors and risk factors for impaired donor site wound healing in this population. In this study, we evaluated the relationship among delayed wound healing, old age, and comorbidities. Hwang et al. reported that the overall complication, perioperative mortality, and donor site complication rates of head and neck free flaps increased significantly with age. The present study also demonstrated that old age was associated with delayed wound healing after RFFF surgery. Chicks et al. evaluated the individual effects of seven different medical conditions in older patients and discussed the increased complication rates associated with an increased number of comorbid conditions [18]. We believe that the correlation between age and delayed wound healing is due to the presence of associated medical problems in patients, the inability to handle the metabolic stresses of surgery, diminished functional capacities of all vital organs in older patients, and insufficient healing; this was also reported by Wester et al. [19].

The prevalence of chronic diseases, such as hypertension and diabetes, increases with age. Hypertension is characterized by the chronic elevation of arterial blood pressure in the arteries (≥140/90 mmHg). Previous studies have shown that hypertension is significantly associated with free flap complications [20]. Ahmed et al. demonstrated that patients with hypertension have a higher risk of prolonged wound discharge than their normotensive counterparts. The precise mechanisms underlying the delay in wound healing are unknown, and pharmacological prophylaxis methods, including antithrombotic and antiplatelet agents, interfere with the coagulation or clotting cascade, potentiating the delayed wound healing effect [21]. Although the underlying mechanisms remain unclear, clinical studies have revealed a relationship between pathological scar development and hypertension [22]. The three main phases of wound healing include inflammation, proliferation, and remodeling. If systemic hypertension can change any of these phases, this suggests that hypertension can be involved in abnormal wound healing. Graf et al. described that elevated blood pressure leads to the activation of the renin–angiotensin–aldosterone system, which is followed by TGF-β/Smad3 signaling activation and the increased local inflammation, extracellular matrix production, and fibrosis seen in hypertensive heart disease [23]. During the inflammatory phase, the metabolic demands of the cells increase. However, the metabolite substrate levels decrease because of the compression caused by interstitial hypertension [24]. These effects can lead to tissue hypoxia. Oxygen promotes angiogenesis and collagen synthesis, produces growth factors and reactive oxygen species, and ensures the efficient functioning of leukocytes and fibroblasts [25]. Any impairment in the oxygen supply can delay healing. Although no studies have shown a strong cause-and-effect relationship between hypertension and abnormal wound healing, hypertension is likely to be a risk factor for delayed wound healing. This information is clinically useful as it suggests that screening patients with hypertension may help prevent delayed wound healing and apply postsurgical wound care strategies. Further in vitro and in vivo studies are required to explore the relationship between hypertension and delayed wound healing.

The superficial position of the sensory branch of the radial nerve is vulnerable to injury during harvesting of the radial forearm flap [26]. Damage to or exposure to these sensory nerves may result in sensory changes after surgery, manifesting as painful neuroma, paresthesia, or cold intolerance [27]. Preventing neuropathic symptoms is important because they can cause complaints in most patients. However, determining the exact source of neuropathic pain in these nerves remains challenging. Although the lateral antebrachial cutaneous nerve is usually dissected during flap elevation, this does not necessarily lead to neurogenic complications. Iatrogenic nerve injury may trigger molecular changes in nociceptive neurons, which eventually become abnormally sensitive and develop spontaneous pathological activity [28]. Additionally, even if there is no direct injury, it is possible to cause symptoms due to the exposed nerves and skin grafts, which are used for donor site coverage and often overlie the nerves, possibly causing nerve irritation. In this study, we found a mean incidence of 13.9% for paresthesia around the donor site and a mean incidence of 10.8% for pain. The incidence of paresthesia was significantly higher in patients with diabetes. In contrast, when skin grafting was performed with ADM, the incidence of paresthesia was significantly lower.

Neuropathy is a common complication in patients with diabetes. Owing to the variety of underlying pathogenetic mechanisms and the resulting diversity of clinical presentations, they are considered diabetic neuropathies rather than a single entity of diabetic neuropathy. Diabetic peripheral neuropathy (DPN) causes symptoms such as numbness, pain, and inability to feel heat or cold, which can cause discomfort and require additional management. As DPN is an independent risk factor that threatens the limbs, which includes postoperative infection and foot ulcers, it is essential to identify patients with DPN who undergo foot and ankle surgeries [29]. Clearly, neuropathy in diabetes offers a specific and important diagnostic challenge for clinicians and plays a definitive role in differential diagnosis. The high rate of paresthesia among patients with diabetes after RFFF may be a symptom related to diabetic neuropathy but may also be due to pressure applied to the nerve because of contractures that occur during wound healing after skin grafting [30]. Although still debated, peripheral nerves in patients with diabetes can easily be damaged by external pressure or tightness. Many studies have investigated the relationship between diabetes and carpal tunnel syndrome (CTS) based on the assumption that diabetes can make the peripheral nerves susceptible to entrapment [30], and several mechanisms, including mechanical compression and microvascular insufficiency, have been suggested to cause CTS [31]. Nonetheless, the findings of the present study suggest that it may be advisable to use an artificial dermal substitute—ADM—to avoid paresthesia.

Wound shrinkage and graft contracture have been reported at the donor site of an RFFF, resulting in functional limitations. Donor site morbidities, such as excessive scarring, motion-limiting contractures, and pigmentation, have prompted the exploration of the notion that various ADMs improve donor site outcomes [32]. Therefore, ADM is commonly used with split skin grafts to compensate for the limited range of motion and stiffness, especially near the joint area. Additionally, co-grafting with ADM is known to minimize scar contracture and improve scar quality [33]. Despite an abundance of literature on the benefits of ADM, its effects on neurological complications remain unclear. Neuropathic symptoms at the donor site are likely to be missed or underdiagnosed. However, the incidence rate is relatively high [34]. Harvesting a flap from the forearm could lead to iatrogenic nerve damage, and aside from the iatrogenic nerve damage during harvesting, the skin graft may also be a source of neuropathic symptoms at the donor site. A skin graft is used for donor site closure, which often overlies the nerves in this area, possibly causing nerve irritation [35]. Therefore, several techniques have been described to manage nerve injuries during surgery and prevent neuropathic pain. In addition to neurectomy and burying of the nerve in the muscle or bone to reduce nerve damage, nerve irritation can also be reduced using ADM with a skin graft. Watfa et al. found that skin sensibility and superficial radial nerve recovery were better in a group that used a dermal substitute (Matriderm™) [36]. Lee et al. reported that the pain sub-score was significantly lower in an STSG with ADM group [37]. An STSG can adhere to the underlying fascia or muscle layer, causing nerve irritation. This is because the amount of dermis is negatively related to the degree of scar contracture [38]. Therefore, by supplementing the dermis with dermal substitutes containing collagen and elastin, the scarring quality is improved. In support of this scenario, little is known about this problem, highlighting the importance of investigating it in future studies.

In a study by De Witt et al., 61% of patients complained of cosmesis and forearm sensibility of the forearm. Furthermore, 30% of patients suffered from sensory changes, and 25% experienced forearm disabilities due to donor site morbidity [34]. However, cosmetic dissatisfaction with donor scars is relative. As the RFFF is often performed as part of oncological head and neck reconstructions, follow-up focuses on survival from the disease or severely disabling complications at the recipient site, including speech and mastication impairment [39]. This is especially true for older patients, where the overall complication rate increases with age because of the medical condition of the patients, longer operative time, inability to handle the metabolic stresses of surgery, and insufficient healing. As stated by Wester et al. [19], it is often advantageous to keep the operation time short, even when it may result in conspicuous scars, rather than opt for a time-consuming operation only for the purpose of a favorable cosmetic result. Therefore, the RFFF is an attractive option for head and neck cancer reconstruction, which is common among older individuals [12]. In this study, the possibility of complications due to scarring at the donor site was fully explained before surgery, and all patients agreed to undergo the surgery. According to the questionnaire, itching sensations and scar irregularities were noted in some patients. However, only three patients received treatment for these reasons, which was not a significant clinical problem. Additionally, no significant factors affecting scar treatment were identified; however, it was not confirmed whether ADM increased scar satisfaction.

The limitations of this study included the small number of patients and the implementation of one skin graft technique instead of two (full-thickness vs. split-thickness). The small sample size increased the likelihood of a type II error, skewing the results and decreasing the power of this study. However, we addressed these limitations through additional research. Second, the severity of hypertension at the time of the RFFF reconstruction was not considered. Thus, although this factor was identified as a predictor of delayed wound healing, we could not provide specific parameters, such as systolic/diastolic pressure, that predispose patients to complications. This limits the potential of our findings to recommend therapeutic interventions for patient optimization before sternal reconstruction. Third, because this was a retrospective study, there was no randomization, and selection bias may have affected the observations and conclusions. There was no preoperative evaluation of diabetic neuropathy in patients with diabetes, and sensory changes that appeared as various symptoms were judged to be collectively referred to as sensory changes without being specifically categorized according to the area or symptoms, which weakened the significance of our results. A more detailed analysis is required in future studies. Further research should focus on the specific mechanisms underlying the effect of hypertension on wounds, the relationship between ADM and sensory changes following surgery, and preventive measures to improve the clinical outcomes of this widely used flap. Nevertheless, the findings of this study laid the foundation for more in-depth prospective studies aimed at characterizing the causes and underlying mechanisms of hypertension, diabetes, and ADM, which contribute to donor site complications after RFFF reconstruction.

## 5. Conclusions

Although the RFFF has disadvantages, it is more commonly used in the reconstruction of head and neck cancers, especially in older patients, because functional rather than cosmetic results are emphasized. In this study, we found that hypertensive patients undergoing RFFF reconstruction are at risk of delayed wound healing and thus should be evaluated carefully. Diabetes may also be a risk factor for paresthesia. However, ADM use reduces the incidence of paresthesia. In conclusion, clinicians should pay particular attention to wound healing strategies in patients with hypertensive RFFFs. The use of ADM with skin grafts is beneficial in terms of sensory changes.

## Figures and Tables

**Table 1:** Baseline characteristics of the patients.

Variable	Subgroup	Statistics (%)
N (%)		67 (100%)
Sex	Female	18 (27%)
	Male	49 (73%)
Age		66.00 (60.00~70.00)
Body mass index		22.90 (20.00~24.79)
Diabetes mellitus		11 (16%)
Hypertension		15 (22%)
Heart disease		3 (4%)
Smoking		8 (12%)
Location	Tongue cancer	12 (18%)
	Buccal cancer	10 (15%)
	Hypopharynx cancer	9 (13%)
	Tonsil cancer	9 (13%)
Metastasis of lymph node		11 (16%)
Chemotherapy		11 (16%)
Follow-up time (month)		8.00 (6.00~10.50)

Continuous variables are expressed as median (interquartile range). Categorical variables are expressed as sample numbers (%; 95% confidence interval, %).

**Table 2:** Surgery-related characteristics of the patients.

Variable	Subgroup	Statistics (%)
Forearm direction used in RFFF	left	52 (78%)
	right	15 (22%)
Skin graft size (cm^2^)		60.00 (42.90~87.30)
Use of acellular dermal matrix	not used	6 (9%)
	CGderm	16 (24%)
	megaderm	23 (34%)
	metriderm	22 (33%)

RFFF: radial forearm free flap.

**Table 3:** Donor site complication assessment.

Complications	Number of Events (%)
Early complications	
Seroma	4/67 (6%)
Hematoma	10/67 (15%)
Delayed wound healing (taking more than 3 weeks to complete healing)	25/67 (37%)
Wound infection	3/67 (4%)
Wound dehiscence	1/67 (1%)
Late complications	
Persistent wrist stiffness	3/67 (4%)
Hand swelling	3/67 (4%)
Reduced hand strength	2/67 (3%)
Paresthesia	9/67 (13%)
Itching sensation and pain (scar)	7/67 (10%)
Scar treatment history	5/67 (7%)

**Table 4:** Wilcoxon rank-sum test, Fisher’s exact test, and significance probability of delayed wound healing and paresthesia in multiple variables.

	Sex	Age	DM	HTN	Smoking	ADM
Delayed wound healing	0.123	0.05	0.268	0.05	1.000	0.653
Paresthesia	0.566	0.891	0.023	0.064	0.244	0.024

**Table 5:** Effect of the clinic variables on delayed skin graft healing.

Variable	Subgroup	N	Case N (%)	Univariable Analysis	*p* Value
				OR (95% CI)	
Sex	F	18	1 (1%)	Ref.	
	M	49	13 (19%)	5.687 (0.618–52.338)	0.125
Age				1.187 (1.045–1.350)	0.009
Body mass index				0.912 (0.741–1.124)	0.389
Diabetes mellitus		20	11 (16%)	2.700 (0.633–11.509)	0.179
Hypertension		27	18 (54%)	9.000 (1.958–41.365)	0.005
Heart disease		5	2 (3%)	0.808 (0.066–9.822)	0.867
Smoking		8	3 (4%)	0.982 (0.195–4.941)	0.982
Metastasis of lymph node		14	7 (10%)	0.914 (0.212–3.939)	0.904
Chemotherapy		20	7 (10%)	0.914 (0.212–3.939)	0.904
Arm direction	Left	52	20 (30%)	Ref.	
	Right	15	5 (7%)	2.864 (0.415–19.773)	0.286
Skin graft size (cm^2^)				1.005 (0.982–1.028)	0.675
Postoperative day				1.088 (0.931–1.272)	0.290
Use of acellular dermal matrix		61	6 (9%)	0.550 (0.094–3.201)	0.506

Continuous variables are expressed as median (interquartile range). Categorical variables are expressed as sample numbers (%; 95% confidence interval, %).

**Table 6:** Effect of clinic variables on paresthesia.

Variable	Subgroup	N	Case N (%)	Univariable Analysis	*p* Value
				OR (95% CI)	
Sex	Female	18	1 (1%)	Ref.	
	Male	49	8 (12%)	25,141,041.956 (0.000–Inf)	0.994
Age				1.028 (0.904–1.170)	0.673
Body mass index				0.885 (0.662–1.184)	0.412
Diabetes mellitus		20	7 (10%)	13.714 (1.311–143.446)	0.029
Hypertension		25	7 (10%)	8.400 (0.828–85.215)	0.072
Heart disease		5	2 (3%)	3.625 (0.264–49.705)	0.335
Smoking		13	4 (6%)	3.467 (0.456–26.372)	0.230
Metastasis of lymph node		18	3 (4%)	1.917 (0.270–13.631)	0.516
Chemotherapy		18	4 (6%)	1.917 (0.270–13.631)	0.516
Arm direction	Left	52	9 (13%)	Ref.	
	Right	15	0 (0%)	0.000 (0.000–Inf)	0.995
Skin graft size (cm^2^)				1.032 (1.000–1.066)	0.053
Post opertive day				1.109 (0.961–1.281)	0.158
Use of acellular dermal matrix		61	4 (6%)	0.071 (0.008–0.613)	0.016

Continuous variables are expressed as median (interquartile range). Categorical variables are expressed as sample numbers (%; 95% confidence interval, %).

## Data Availability

The raw data supporting the conclusions of this article will be made available by the authors upon request.
